# A plasma 9-microRNA signature for lung cancer early detection: a multicenter analysis

**DOI:** 10.1186/s40364-025-00787-x

**Published:** 2025-05-16

**Authors:** Elisa Dama, Tommaso Colangelo, Roberto Cuttano, Rafal Dziadziuszko, Thomas Dandekar, Piotr Widlak, Witold Rzyman, Giulia Veronesi, Fabrizio Bianchi

**Affiliations:** 1 Unit of Cancer Biomarkers, Fondazione IRCCS Casa Sollievo della Sofferenza, Vale Padre Pio, 7, San Giovanni Rotondo, 71013 Italy; 2 Department of Oncology and Radiotherapy, Faculty of Medicine, Medical University of Gdańsk, Gdańsk, Poland; 3 Department of Bioinformatics, Biocenter, University of Würzburg, Würzburg, Germany; 4 2nd Department of Radiology, Medical University of Gdańsk, Gdańsk, Poland; 5 Department of Thoracic Surgery, Faculty of Medicine, Medical University of Gdańsk, Gdańsk, Poland; 6 Department of Thoracic Surgery, IRCCS San Raffaele Scientific Institute, Milan, Italy

**Keywords:** Lung cancer, Early diagnosis, MicroRNA, Liquid biopsy, Machine learning

## Abstract

Lung cancer remains the leading cause of cancer-related deaths worldwide. Low-dose computed tomography (LD-CT) screening, combined with effective minimally invasive molecular testing such circulating microRNA, has the potential to reduce the burden of lung cancer. However, their clinical application requires further validation, including studies across diverse patient cohorts from different countries. In this study, we propose a signature of 9 circulating miRNAs derived from a robust multi-platform workflow with a multi-center design, for a total of 276 lung cancer and 451 non-cancer controls, based on the data from two European LD-CT screening cohorts (Poland and Italy). The classification performance of the signature was stable in the two screening cohorts, with AUC=0.78 (SE, 76%; SP, 67%; ACC=70%), and AUC=0.75 (SE, 82%; SP, 68%; ACC=71%) in the Polish and Italian cohorts, respectively. The diagnostic accuracy of the signature was remarkably independent of age, gender, smoking (status and intensity), nodule size, and density. Additionally, the signature demonstrated strong performance in detecting stage I lung cancer, with AUC=0.76 (95%CI: 0.68-0.84), and 0.69 (95%CI: 0.49-0.89) in the Polish and Italian cohorts respectively, with a prediction ability of 63-73%. The signature’s ability to discriminate benign nodules was satisfactory, with AUC=0.71 (95%CI: 0.58-0.84). The proposed panel of 9 circulating miRNAs provides a robust and precise diagnostic tool to substantially advance the effectiveness of the LD-CT screening program.

**Supplementary Information:**

The online version contains supplementary material available at 10.1186/s40364-025-00787-x.

## To the Editor

Lung cancer (LC) is the most common cancer worldwide, with 2.5 million diagnoses and 1.8 million deaths annually [1]. LD-CT screening reduces LC mortality by 20–30% through early detection [2, 3]. However, concerns over cost, radiation exposure, false positives, and overdiagnosis remain [4]. Tumor biomarkers could improve LD-CT specificity and reduce false positives. Two blood microRNA signatures, the MSC classifier and miR-Test, have been validated in large screening studies [5, 6], but clinical implementation is lacking due to limited multi-cohort validations. To address this, we developed a multi-platform workflow and tested a robust panel of circulating miRNA (c-miR) LC biomarkers in two European LD-CT screening cohorts.

### Meta-signature identification

**Fig. 1:**
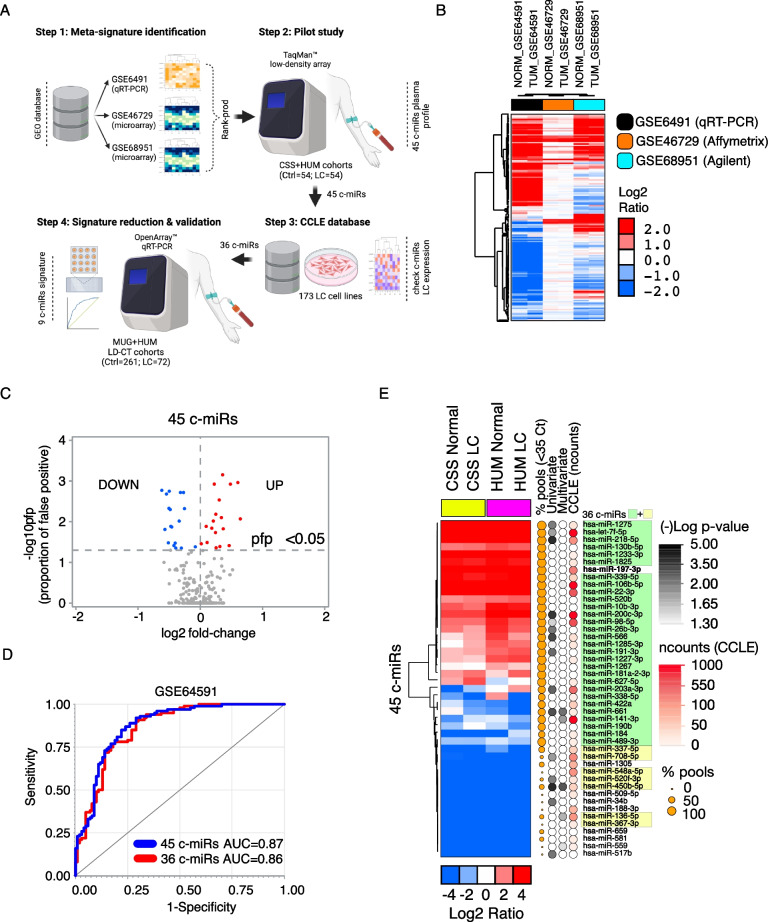
**A** Schematic representation of the study. **B** Hierarchical clustering analysis of the 321 c-miRs commonly identified in all 3 c-miR expression datasets (GSE64591, GSE46729, GSE68951). Data (arrays) were median centered. Colors are as per the legend. **C** Volcano plot for the 321 c-miRs common to the 3 datasets GSE64591, GSE46729, GSE68951. Log2 fold-change and –log10 proportion of false positive (pfp) are reported, as per RankProd non-parametric method. Each dot represents one miRNA. Selected 45 c-miRs differentially expressed (pfp<0.05) are highlighted in red (upregulated) or in blue (downregulated) in tumor vs. normal samples; **D** ROC curves and AUC, for the model including 45 c-miRs and 36 c-miRs selected for the further testing (STEP-4), applied to the IARC dataset (GSE64591); **E **Hierarchical clustering analysis of the 45 c-miRs expression (data were median-array-centered) in the pools (*N*=6) of samples (*N*=108) collected at IRCCS Casa Sollievo della Sofferenza Hospital (CSS) and Humanitas Research Hospital (HUM). Colors are as per the legend. On the right, bubbles represent the different criteria applied (as per the legend) to identify the 36 c-miRs. Highlighted in green, the 29 c-miRs selected (reliable detection in Step 2 analysis), and in yellow, the remaining 7 c-miRs selected in Step 3 and GSE64591 analysis. In bold, has-miR-197-3p which is included in the panel of 6 previously identified housekeeping c-miRs

To develop a robust c-miR diagnostic signature, we implemented a four-step strategy (Fig. 1A, Figure S1; Supplemental Methods). In STEP-1, we performed an in-silico analysis of publicly available datasets containing expression data for hundreds of c-miRs (GSE64591, GSE46729, GSE68951) from 150 lung cancer (LC) patients and 136 controls (Table S1), identifying 321 shared c-miRs (Fig. 1B; Table S2). Using RankProd meta-analysis to accommodate screening platforms variability, we found 45 differentially expressed c-miRs (pfp <0.05) between LC and controls (Fig. 1C; Table S2), achieving an AUC=0.87 (95%CI: 0.83–0.92) in the largest dataset (GSE64591, N=200) (Fig. 1D).

In STEP-2, we measured 45 c-miRs expression in plasma samples from 54 LC patients and 54 controls, that we pooled in 6 LC and 6 CTRL samples (Table S3), ranking them based on reliable qRT-PCR data (i.e., <35 Ct in at least 50% of pools), refining the list to 29 c-miRs markers (Fig. 1E; Tables S4). In STEP-3, we revisited the 45 c-miRs intracellular expression in 173 LC cell lines (CCLE) to *(i)* recover c-miRs that may have been underrepresented in the limited-sized cohort analyzed in STEP-2, and *(ii)* support the LC specificity of our final 9-c-miR diagnostic signature, and analyzed the effect of 45 c-miRs on LC risk in GSE64591 dataset. As a result, we refined the signature to 36-c-miR (29+7 c-miRs) achieving an AUC=0.86 (0.81–0.91) (Fig. 1D-E, Figure S1, Table S4).

### Multi-center analysis of c-miR signature in LD-CT screening cohorts

**Fig. 2:**
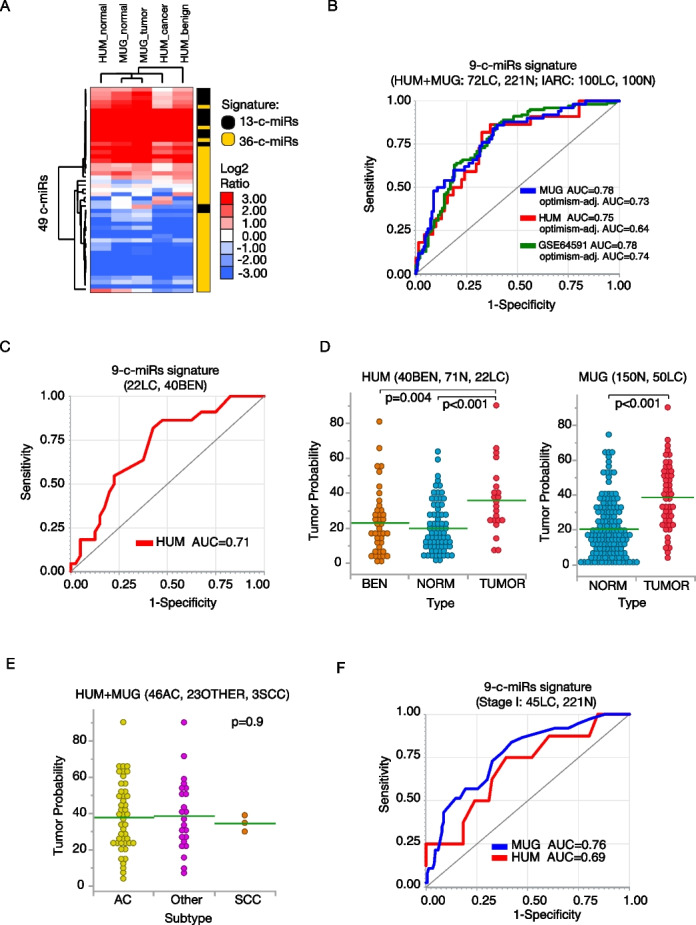
**A** Hierarchical clustering analysis of the median 36 c-miRs and 13 c-miRs (external signature) expression on real-word multi-center LD-CT screening cohorts of high-risk subjects (Step 4 analysis). Colors are as per the legend. **B** ROC curves, AUC, and optimism-adjusted AUC (200 bootstrap) for the 9-c-miR model in the following cohorts: MUG screen-detected lung cancer (LC) and normal controls (N), HUM screen-detected lung cancer (LC) and normal controls (N), and IARC GSE64591 lung cancer (LC) and normal controls (N). **C** ROC curve and AUC for the 9-c-miR model applied to the HUM cohort: screen-detected lung cancer (LC) and benign (BEN). **D** Distribution of the probability of having lung cancer (tumor predicted probability) using the 9-c-miR model in MUG and HUM cohorts; green lines represent the mean values. **E** Distribution of tumor predicted probabilities using the 9-c-miR model in the various LC subtypes (adenocarcinoma, AC; squamous cell carcinoma, SCC; and other subtypes, Other); green lines represent the mean values.** F** ROC curves and AUC for the 9-c-miR model applied to stage I disease only in MUG and HUM cohorts

In Step 4, we evaluated the 36-c-miR signature in a multi-center LD-CT screening cohort (72 LC cases, 261 controls) from Poland (‘MUG’ cohort) and Italy (‘HUM’ cohort) (Table S5; Supplemental Methods). We also included other 13 c-miRs (aka miR-Test) we previously showed to accurately diagnose LC [6]. Using OpenArray™ qRT-PCR, we profiled plasma samples in a single batch, minimizing technical variability (Fig. 2A). A stepwise approach in the MUG cohort resulted in a further reduced 9-c-miR signature (along with 3 additional c-miRs used for normalization; see Supplemental Methods) with an AUC of 0.78 (SE, 76%; SP, 67%; ACC=70%) (Fig. 2B; Table S6). Testing in the HUM cohort yielded an AUC=0.75 (SE, 82%; SP, 68%; ACC=71%) (Fig. 2B; Table S6), while independent testing in the GSE64591 dataset (with has an adequate sample size) yield an AUC=0.78 (95%CI:0.71–0.84) (Fig. 2B). The performance of the 9-c-miR signature was also compared to the miR-test, which served as a benchmark (Figure S2; Supplemental Methods). Remarkably, the 9-c-miR signature also discriminated LC from benign nodules (AUC=0.71, 0.58–0.84; Fig. 2C) and significant separation of tumor predicted probability between LC and controls (Fig. 2D), but not across LC subtypes (Fig. 2E).

### Multivariate and subgroup analysis

Multivariable models confirmed a significant association with LC risk (OR=1.27, ‘MUG’; OR=1.5, ‘HUM’; Table S7). The odds ratios (ORs) remained consistent between univariate and multivariable models for all significant factors (i.e., 9-c-miR probability and nodule size, Table S7). Subgroup analyses showed robust diagnostic performance across all subsets (Table S8). Every 5% increase in predicted probability was associated with 29% and 25% increase odds of having stage I LC in MUG (*p*<0.0001) and HUM cohorts (*p*=0.0224) (Table S8), respectively. The signature achieved an AUC=0.76 (0.68–0.84) in MUG and AUC=0.69 (0.49–0.89) in HUM (Fig. 2F), correctly identifying 73% of stage I LC cases in the MUG cohort (IA, *N*=32; IB, *N*=5) and 62.5% in the HUM cohort (IA, *N*=6; IB, *N*=2) at the cut-off maximizing the Youden index (Table S9).

## Discussion

Serum and plasma microRNA assessment offers a promising complement to LD-CT lung cancer (LC) screening by refining eligibility and improving nodule evaluation. However, prior studies have identified c-miR signatures with minor overlaps and suboptimal during validation due to variability in study design and analytical methods [7]. In this study, we identified a robust 9-c-miR signature with ~70% accuracy, 76%−82% sensitivity, and ~67% specificity in multi-center cohorts, independent of established LC risk factors. Our signature overlaps with previously proposed miRNA panels [5, 6, 8–10] and was identified using stringent meta-analytic and machine learning approaches. While our current findings demonstrates reliability of 9-c-miR test [11] and support the use of the 9-c-miR signature to enhance the accuracy of LD-CT in lung cancer screening, as also previously suggested [5], its potential diagnostic utility in evaluating indeterminate lung nodules cannot be excluded and is currently being further investigated.

A key strength of the study is the inclusion of both clinical and LC screening cohorts with diverse clinical and pathological characteristics, as well as variability in sample handling and screening platforms [12], which enhances the robustness of the findings. A limitation is the limited sample size of LC screening cohorts, which prevented splitting the samples for independent validation of the 9-c-miR test.

## Supplementary Information

Supplementary Material 1.

Supplementary Material 2.

Supplementary Material 3: Figure S1. Study flow-chart with cohorts, analyses, and main results.

Supplementary Material 4: Figure S2. ROC curves, AUC for the 13-c-miRs model in the following cohorts: MUG screen-detected lung cancer (LC) and normal controls (N), HUM screen-detected lung cancer (LC) and normal controls (N).

## Data Availability

The dataset supporting the conclusions of this article is available in the Gene Expression Omnibus repository, with the following accession number: GSE279209.

## Abbreviations

LC: Lung cancer
LD-CT: Low-dose computed tomography
CTC: Circulating tumor cells
ctDNA: Circulating tumor DNA
c-miR: Circulating miRNA
HUM: Humanitas Research Hospital, Milan
MUG: Medical University of Gdansk
SF: Scaling factor
FC: Fold-change
PFP: Proportion of false positive
AUC: Area under the curve
ACC: Accuracy
SE: Sensitivity
SP: Specificity
PPV: Positive predictive value
NPV: Negative predictive value
OR: Odds ratios
EV: Extracellular vesicle
NSCLC: Non-small cell lung cancer
