# Supplementary material for: A plasma 9-microRNA signature for lung cancer early detection: a multicenter analysis
Source: Biomark Res. 2025 May 16;13:74. doi: 10.1186/s40364-025-00787-x (PMC12085043; doi:10.1186/s40364-025-00787-x)
Supplement: Supplementary file 1 — Supplementary Material 1. [file 40364_2025_787_MOESM1_ESM.docx]

**Supplemental methods**

**Study design**

We structured the identification of the diagnostic c-miRs signature into four main Steps, as depicted in Figure 1A: Step 1 – meta-signature identification; Step 2 – pilot study; Step 3 – cancer cell lines; Step 4 – signature reduction and testing (multi-center European screening study).

**Meta-signature identification**

C-miRs expression analysis was performed by using the following publicly available datasets: GSE64591, GSE46729, and GSE68951 (Gene Expression Omnibus database, https://www.ncbi.nlm.nih.gov/geo/query/acc.cgi). Plasma or serum samples for a total of 150 lung cancer and 136 normal controls were profiled through TaqMan Human MicroRNA Array A + B Card Set v3.0, Applied Biosystems (GSE64591), Affymetrix Multispecies miRNA-1 Array, Thermo Fisher Scientific (GSE46729), and Agilent-031181 Unrestricted Human miRNA V16.0 Microarray, Agilent Technologies, Inc. (GSE68951). Non-parametric RankProd method [1] was applied to combine datasets from different origins (meta-analysis) and identify differentially expressed c-miRs (pfp<0.05) between lung cancer and normal controls (meta-signature).

**Pilot and testing cohorts selection**

After approval from the Institutional Review Board (Medical University of Gdansk approval numbers NKEBN/42/2009 and NKBBN/376/2014; and Humanitas Clinical and Research Center approval number CE Humanitas ex DM 390/18; Fondazione IRCCS Casa Sollievo della Sofferenza approval number BIO-POLMONE - V1.0_08 Giu 16), informed consent was obtained from all the participants. Additionally, our study was conducted in accordance with the Declaration of Helsinki.

Pilot study cohort. A preliminary detection analysis of c-miRs composing the meta-signature was performed in a clinical cohort, including retrospectively collected plasma samples from Casa Sollievo della Sofferenza Hospital, San Giovanni Rotondo, Italy (N=24 lung cancer, N=24 controls) and Humanitas Research Hospital, Milan, Italy (N=30 lung cancer, N=30 controls). We randomly split the cohort into 6 subsets of lung cancer and 6 subsets of controls, with proportional allocation of samples for each center. Specifically, each subset included 8 samples from Casa Sollievo della Sofferenza Hospital and 10 samples from Humanitas Research Hospital, respectively. TaqMan Low-Density Array™ were used for c-miRs profile (see also below).

Testing cohort - Multi-center European LD-CT screening study*.* Two LD-CT screening cohorts of high-risk subjects (smokers, >30 packs/year; aged, >50 years/old) were enrolled at Humanitas Research Hospital, Milan (HUM, Italy; SMAC-1 trial, NCT04315766) and Medical University of Gdansk (MUG, Poland; MOLTEST-BIS trial), and contributed to the CLEARLY project (https://transcan.eu/output-results/funded-projects/clearly.kl) funded by TRANSCAN-2 (JTC 2016). The screening inclusion criteria and protocols were as previously described [2,3]. Briefly, *(i)* for the HUM cohort, inclusion criteria were: age over 55 years, active smokers or former smokers who had quit within the past 15 years, a smoking history of more than 30 pack-years, and a PLCOm2012 risk threshold greater than 2% over six years; *(ii)* for the MUG cohort, inclusion criteria included age between 50 and 79 years and a smoking history of at least 30 pack-years. Plasma samples were collected from participants who were ultimately diagnosed with lung cancer by LD-CT (referred to as “cases”) and from participants with no CT-detected lung nodules or other cancer-related health issues (referred to as “controls”). All available cases and controls plasma samples were matched by age, sex, and smoking history. Participants with benign nodules were defined based on nodule stability over a two-year period as confirmed by CT scans, or by a documented reduction in nodule size as previously described [4–7].

A total of 72 lung cancers, 221 normal controls, and 40 benign lung lesions were analyzed.

**Plasma collection**

MUG (Poland) protocol: a total of 10 ml of blood was collected in EDTA-containing vacutainer tubes. Of this, 1 ml (2 x 500 µl) of blood was then aliquoted into cryovials and stored at -80°C. The remaining 9 ml of blood was centrifuged at 600 x g for 20 minutes at 4°C to separate it into three layers: i) plasma (top); ii) white blood cells (buffy coat, middle); iii) and erythrocytes (bottom). The plasma supernatant was carefully aspirated and pooled into a centrifuge tube, followed by a second centrifugation at 1500 x g for 15 minutes at 4°C. After this step, 6 x 500 µl of plasma was aliquoted into labeled cryovials and stored at -80°C. The white blood cells (buffy coat) were collected from the initial centrifuge tube and also stored at -80°C.

HUM (Italy) protocol: the first 3 ml of blood drawn from the patient was discarded to prevent potential skin contamination, and was not used for plasma preparation. The remaining blood sample (minimum 4.5 ml) was collected in tubes with 0.129M Na Citrate anticoagulant (BD Vacutainer Sodium Citrate tubes – 363079 – Light blue top). The samples were centrifuged within 2 hours and 30 minutes at 1300g for 10 minutes at room temperature. The plasma was then carefully transferred into a 1.5 ml microtube, ensuring the interface was not touched and then subjected to a second centrifugation at 1300g for 10 minutes at room temperature. After this, the plasma was transferred into a 15 ml Falcon tube by gentle pipetting and then 3 x ~0.4 ml aliquots of plasma immediately dispensed into 0.5 ml Cryobank 2D coded tubes (Thermo Fisher Scientific, Cryobank vials, 2D coded, racked, blue cap; Cat. No. 374025) and placed on dry ice. The aliquots were kept on dry ice at all times before being transferred to a dedicated -80°C freezer for storage. The samples were also shipped on dry ice.

To identify and eventually exclude hemolytic samples that could negatively affect c-miRs profiling [8], we analyzed the hemolysis index as previously described [9]. The absorbance peaks at 414 nm, and 385 nm, along with the hemolysis index were recorded in the database. Samples with an H.I. < 0.2 were flagged as low-hemolytic.

**Qiacube RNA extraction procedure**

Plasma samples were thawed on ice, and RNA was extracted with the commercial miRNeasy mini-Kit (QIAgen™, Hilden, Germany) using the QIAcube™ robot, following the manufacturer´s instructions.

**TaqMan Low-Density Array™ c-miRs analysis**

Pools plasma aliquots for each subset of the pilot study cohort were created and profiled for c-miRs (Step 2) expression using custom TaqMan Low-Density Array™. RNA was reverse-transcribed using the TaqMan Advanced miRNA cDNA Synthesis Kit (Thermo Fisher Scientific). Poly(A) tailing, adapter ligation, RT reaction, and miR-Amp were performed following the manufacturer’s instructions. qRT-PCR was performed following the manufacturer’s instructions (i.e., 95C for 30s, 45 cycles of 95C for 5s, and 60C for 30s) using a Card Custom Advance (Thermo Fisher Scientific) in a QuantStudio 12k Flex (Thermo Fisher Scientific). Raw Ct values were normalized on six housekeeping c-miRs, as previously described [10].

**OpenArray™ qRT-PCR technology for high-throughput c-miRs analysis**

Total RNA was reverse transcribed using the custom RT Primers and components supplied with the TaqMan MicroRNA Reverse Transcription Kit. The pre-amplified samples were assembled with TaqMan OpenArray Real-Time PCR Master Mix and loaded to OpenArray plates using the OpenArray Accufill System and compatible accessories (OpenArray 384-well Sample Plates, OpenArray 384-Well Plate Seals, OpenArray AccuFill System Tips, and QuantStudio 12K Flex OpenArray Accessories Kit). The OpenArray plates used in the study contained custom TaqMan OpenArray Human MicroRNA, allowing us to investigate c-miRs expression in each sample. Real-time PCR reactions were performed using the QuantStudio 12 K Flex Real-Time PCR System and the default parameters of the amplification cycles. Data were quantified by relative threshold cycles (Crt) methods and normalized on miR-16-5p as per the manufacturer’s instructions (Thermo Fisher Scientific). To further compensate for variability, we added additional normalizers by selecting the two miRNAs most correlating with miR-16-5p, from the list of six miRNAs previously described as housekeeping [10](Table S10). Normalization was performed using the following procedure: a scaling factor (SF) was calculated for each sample, by subtracting the average of the three normalizers to a constant value (K=19.079)[10]. Data were then normalized using the formula: Crt normalized=miRNA Crt raw – SF. Normalization was skipped for Crt=40. Batch bias was investigated through analysis of the miRNAs expression distribution across centers (FC analysis, Wilcoxon Rank Sum test; Table S11).

**Signature reduction**

To reduce the size of the signature and select the best set of diagnostic c-miRs, we applied different feature selection methods modeling the odds of lung cancer as a function of miRNAs expression. In detail: A) unconditional logistic regression with stepwise selection (selection parameters: significance=0.2 to enter into the model, significance=0.25 to stay in the model) was applied to the full screening cohort, including lung cancer and normal controls from both HUM and MUG; batch-effect correction was introduced, including the center as an adjustment covariate in the model; B) penalized unconditional logistic regression was applied to the full screening cohort, with Lasso regularization. Cross-validated (10-fold) log-likelihood with optimization (100 simulations) of the tuning penalty parameter was used to control for potential overfitting. The center was introduced in the model as an unpenalized covariate to correct for batch-effect; C) unconditional logistic regression with stepwise selection (selection parameter: significance=0.3 to enter into the model, significance=0.35 to stay in the model) was applied to the MUG cohort; D) penalized unconditional logistic regression was applied to the MUG cohort, with Lasso regularization (10-fold cross-validation, on 100 simulations). Feature selection with other techniques (unconditional logistic regression with elastic-net regularization, diagonal linear discriminant analysis) were applied, without reaching better performance (data not shown). Notably, a stepwise approach with center correction or penalized approach did not allow to obtain signatures with better performance (in terms of Accuracy, Sensitivity, Specificity, PPV and NPV) than the 9-c-miR signature (Table S13-14). Yet, the model based on 13 c-miRs composing our previously derived miR-Test [10] showed and AUC of 0.68 (SE, 66%; SP, 59%; ACC=61%) and AUC of 0.80 (SE, 86%; SP, 59%; ACC=66%) in the MUG and HUM cohorts, respectively (Figure S2).

**Statistical analysis**

Patients and tumors characteristics were presented as number and percentage for categorical variables, and as median with first and third quartiles (Q1;Q3) for continuous variables; we used Fisher’s exact test and Wilcoxon rank sum test to compare differences in distribution of categorical and continuous variables, respectively. Differentially expressed miRNAs across different datasets (meta-signature identification) were identified through the RankProd method, with a non-parametric permutation approach to calculate fold-change and associated proportion of false positive (pfp). Logistic regression was used to model the odds of lung cancer as a function of miRNAs expression (Table S14). Feature selection approaches based on stepwise or Lasso regularization were used to select panels of miRNAs discriminating between lung cancer and normal controls. Internal validation of models was conducted using bootstrapping techniques (200 bootstraps). The diagnostic performance of predictive models was evaluated by calculating the area under the curve (AUC), accuracy (ACC), sensitivity (SE), specificity (SP), positive predictive value (PPV), and negative predictive value (NPV). Youden index was used to identify the optimal threshold. A value of p less than 0.05 was considered statistically significant.

Of note, in STEP-2, we were unable to apply the logistic regression model with 45 c-miRs to GSE46729 and GSE68951 datasets due to convergence issues and overfitting, which were exacerbated by small sample sizes (24 LC vs. 24 normal in GSE46729, and 26 LC vs. 12 normal in GSE68951). Additionally, pooling all three datasets was not feasible due to differences in the profiling platforms used. While a RankProd approach could be applied across datasets, it is not compatible with logistic model implementation.

All statistical analyses were performed using SAS software, version 9.4 (SAS Institute, Inc., Cary, NC), and R 3.3.1 (R Core Team, 2016).

**Reference:**

1. Hong F, Breitling R, McEntee CW, Wittner BS, Nemhauser JL, Chory J. RankProd: a bioconductor package for detecting differentially expressed genes in meta-analysis. Bioinformatics. 2006;22:2825–7.

2. Antonicelli A, Muriana P, Favaro G, Mangiameli G, Lanza E, Profili M, et al. The Smokers Health Multiple ACtions (SMAC-1) Trial: Study Design and Results of the Baseline Round. Cancers. 2024;16:417.

3. Ostrowski M, Marjański T, Dziedzic R, Jelitto-Górska M, Dziadziuszko K, Szurowska E, et al. Ten years of experience in lung cancer screening in Gdańsk, Poland: a comparative study of the evaluation and surgical treatment of 14 200 participants of 2 lung cancer screening programmes. Interactive CardioVascular and Thoracic Surgery. 2019;29:266–74.

4. Maisonneuve P, Bagnardi V, Bellomi M, Spaggiari L, Pelosi G, Rampinelli C, et al. Lung Cancer Risk Prediction to Select Smokers for Screening CT—a Model Based on the Italian COSMOS Trial. Cancer Prevention Research. 2011;4:1778–89.

5. Maisonneuve P, Casiraghi M, Bertolotti R, Rampinelli C, Muriana P, Spaggiari L, et al. P1.04-03 Independent Validation of the Maisonneuve Lung Cancer Risk Model to Optimize Screening Interval in High-risk Individuals. Journal of Thoracic Oncology. 2022;17:S102.

6. Gould MK, Donington J, Lynch WR, Mazzone PJ, Midthun DE, Naidich DP, et al. Evaluation of Individuals With Pulmonary Nodules: When Is It Lung Cancer? Chest. 2013;143:e93S-e120S.

7. MacMahon H, Naidich DP, Goo JM, Lee KS, Leung ANC, Mayo JR, et al. Guidelines for Management of Incidental Pulmonary Nodules Detected on CT Images: From the Fleischner Society 2017. Radiology. 2017;284:228–43.

8. Marzi MJ, Montani F, Carletti RM, Dezi F, Dama E, Bonizzi G, et al. Optimization and Standardization of Circulating MicroRNA Detection for Clinical Application: The miR-Test Case. Clinical Chemistry. 2016;62:743–54.

9. Appierto V, Callari M, Cavadini E, Morelli D, Daidone MG, Tiberio P. A Lipemia-Independent Nanodrop ^®^ -Based Score to Identify Hemolysis in Plasma and Serum Samples. Bioanalysis. 2014;6:1215–26.

10. Montani F, Marzi MJ, Dezi F, Dama E, Carletti RM, Bonizzi G, et al. miR-Test: A Blood Test for Lung Cancer Early Detection. JNCI: Journal of the National Cancer Institute [Internet]. 2015 [cited 2023 Dec 9];107. Available from: https://academic.oup.com/jnci/article-lookup/doi/10.1093/jnci/djv063
