# Supplementary figures and images for: A plasma 9-microRNA signature for lung cancer early detection: a multicenter analysis
Source: Biomark Res. 2025 May 16;13:74. doi: 10.1186/s40364-025-00787-x (PMC12085043; doi:10.1186/s40364-025-00787-x)

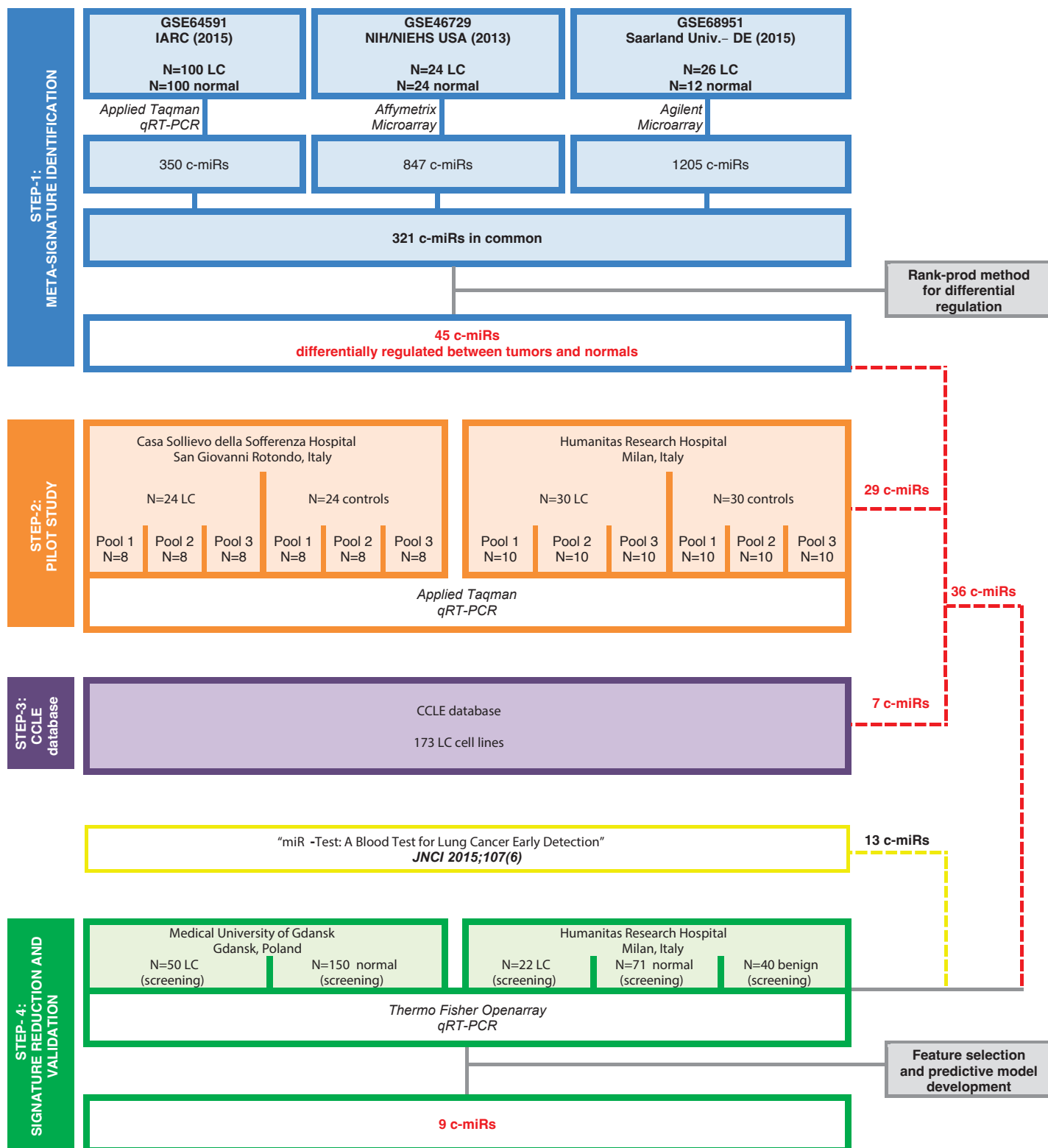

Figure S1

Supplement: Supplementary file 3 — Supplementary Material 3: Figure S1. Study flow-chart with cohorts, analyses, and main results. [file 40364_2025_787_MOESM3_ESM.pdf]

A

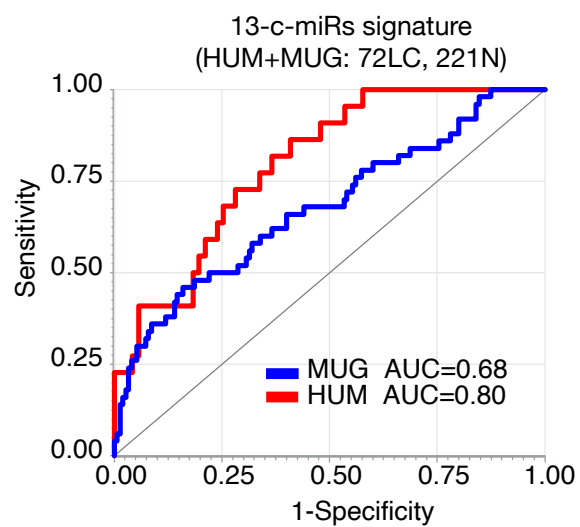

Figure S2

Supplement: Supplementary file 4 — Supplementary Material 4: Figure S2. ROC curves, AUC for the 13-c-miRs model in the following cohorts: MUG screen-detected lung cancer (LC) and normal controls (N), HUM screen-detected lung cancer (LC) and normal controls (N). [file 40364_2025_787_MOESM4_ESM.pdf]
